# Medical Miscellanies

**Published:** 1816-10

**Authors:** 


### PART IV.

### MEDICAL MISCELLANIES.

Quicquid agunt Homines.

*Cessation of the Pulse at both Wrists for Thirty Hours, with ultimate Recovery.*—One of the Editors was lately requested to see a woman who was reported to be very ill. He found her with some pyrexia, heat on the skin, great irritability of the stomach, inability to retain any food or drink, violent pain in the left hypo-

---

chondrium, and a *spontaneous* salivation. Pressure on the region of the stomach, and on every part of the abdomen, gave pain; her eyes were yellow; her tongue thickly furred; her stools scanty, dark, and scybalous. A pretty large dose of calomel was given, and a blister applied to the principal seat of pain. She continued in great suffering for two days, and, notwithstanding that frequent doses of calomel were given, no proper fæcal matter could be procured. On the third day, the pain suddenly ceased, the pulse at the wrists totally disappeared, and the extremities became cold. She was ordered a cordial mixture with carbonate of ammonia, and apprehension was entertained that mortification had taken place internally. No alteration occurred, nor the slightest return of pulse for thirty hours; when a *mercurial* ptyalism came on; copious, bilious, and fæculent stools were passed; and in two days she was down stairs with a ravenous appetite.

Dr. WEINHOLD, of Maresburgh, recommends the application of Galvanism through the medium of mercury, soda, potass, and ammonia in several disorders of the retina and corpus vitreum. For the purpose of applying the galvanic fluid to the human eye, through the medium of liquid and solid medicines, he has in the course of three years tried nearly a hundred different remedies, intending to publish the results, as they are alike important both for the physiologist and the medical practitioner; but as yet he has been prevented, partly by the pressure of professional occupations, and partly by the unfavourable situation of the book-trade. In a pamphlet, entitled "*Cure of a destroyed Eye,*" Meissen, 1813, he has, however, given some hints how liquids enclosed in glass cylinders are to be applied as a medium of galvanic applications to the human morbid eye: and in another, entitled "*On an Epidemic Disorder of the Eye,*" Dresden, 1815, he shews how mercury is to be employed for the same purpose. He now adds, that besides mercury, formic acid and ammonia, solutions of soda, potass, and ammonia, combined with an adequate internal treatment, served to obviate a beginning dyscracy of the hyaloidea, the organ secreting the vitreous humour, and that they have, under similar treatment, cured many an amaurosis which had already begun to form.

*The Badé Sumoom, or pestilential Wind of the Desert in Beloochistan.* (Pottinger's Travels.)—" Its effects on the human frame were related to me by those who had been eye-witnesses of them, as the most dreadful that can be imagined; the muscles of the unhappy sufferer become rigid and contracted; the skin shrivels; an agonizing sensation, as if the flesh was on fire, pervades the whole frame; and, in the last stage, it cracks into deep gashes, producing hæmorrhage that quickly ends his misery. In some instances life is annihilated instantaneously, and in others the unfortunate victim lingers for hours, or perhaps days, in the excruciating tortures I have described. To render this terrible scourge still more baneful, its approach is seldom or ever foreseen; and, among all the Belooches with whom I have conversed regarding it, no one asserted more, than that they had heard it was indicated by an unusual oppression in the air, and a degree of heat that affected the eyes: the precaution then adopted is to cover themselves over, and lie prostrate on the earth. A curious fact is established by this custom, that any cloth, however thin, will obviate the deleterious effects of the Badé Sumoom on the human body." p. 136.

The tantalizing effects of that remarkable phænomenon, the Mirage, on the thirsty travellers, are thus delineated: — "The *Suhrab*, or water of the desert, floated all around us, as though it were mocking our distress by its delusive representation of what we so eagerly thirsted for; the absence of which, I can affirm with perfect confidence, from my individual experience, to be the most insupportable of all the wants of what are termed absolute necessaries of life. A person may endure, with patience and hope, the pressure of fatigue and hunger, heat or cold, and even a total deprivation of natural rest for a considerable length of time; but to be scorched under a burning sun, to feel your throat so parched and dry that you respire with difficulty, to dread moving your tongue in your mouth from the apprehensions of suffocation which it causes, and not to have the means of allaying those dreadful sensations, are, in my ideas, the extreme pitch of a traveller's calamities. The Suhrab, of which I have just spoken, is said to be caused by the rarefaction of the atmosphere from extreme heat; and, which augments the delusion, it is most frequently in hollows where water might be expected to lodge. I have seen bushes and trees reflected in it with as much accuracy as though it had been the face of a clear and still lake; and once, in the province of Kerman, in Persia, it seemed to rest, like a sheet of water, on the face of a hill, at the foot of which my road lay, exhibiting the summit, which did not overhang it in the least degree, by a kind of unaccountable refraction. This phænomenon is, however, very uncommon; and the Persians, who were travelling with me, attributed it to exhalations from saline particles, with which the hill abounds." *Travels*, p. 185.

*Premature Interment among the Moors of Barbary.* — "As, according to their religion, they cannot think the departed happy till they are under ground, they are washed instantly, while yet warm; and the greatest consolation the sick man's friends can have, is to see him smile while this operation is performing, as they look on that as a sign of approbation in the deceased, not supposing such appearance to be a convulsion, occasioned by washing and exposing to the cold air the unfortunate person, before life has taken its final departure. This accounts for the frequent instances that happen of people being buried alive: many of the Moors say a third of the people are lost in this manner." *Narrative of a Ten Years' Residence in Tripoli.*

The following "*Chemical Analysis of the Moira Saline Water*, (recently discovered at the distance of two miles from Ashby de la Zouch, Leicestershire) by Mr. ACCUM, of London," has been officially transmitted to us for insertion in our Journal.

| "One wine gallon of the water contains, of | *Grains.* |
|---|---|
| Muriate of soda | 1838 |
| ——— of magnesia | 210 |
| ——— of lime | 172 |
| Carbonate of iron | 18 |
| Sulphate of lime | 96 |
| Sulphate of soda | 130 |
| Carbonate of lime | 42 |
| | 2506." |

The name of Mr. Accum will be deemed a sufficient pledge for the accuracy of this analysis. We shall, elsewhere, speak of the medicinal virtues and employment of this strongly-impregnated saline spring.—EDITORS.

*Original Recipe for the Black Drop.*—Take half a pound of opium sliced, three pints of good verjuice, one ounce and a half of nutmegs, half an ounce of saffron. Boil them to a proper thickness, then add a quarter of a pound of sugar and two spoonfuls of yeast. Set the whole in a warm place near the fire for six or eight weeks, then place it in the open air until it become a syrup. Lastly, decant, filter, and bottle it, adding a little sugar to each bottle." These ingredients, when properly mixed, should yield about two pints of the strained liquor. One drop of it is supposed to be equal to three drops of the tincture of opium of the London Pharmacopœia." "The black drop is a most excellent preparation of opium, and highly deserving of a place in our Pharmacopœias. From the quantity of acid in its composition, it will often stay upon the stomach when other preparations will not; and, in the hands of a judicious physician, may, therefore, be usefully applied."—*Dr. Armstrong's Practical Illustrations of Typhus, &c.*

Dr. CLOUGH, Physician-Accoucheur to the St. Marylebonne General Dispensary, and to the Endeavour or Benevolent Society for delivering poor Women at their own Habitations, &c. &c. commences his Autumnal Course of Lectures on the Science and Practice of Midwifery, including the Diseases of Women and Children, at his Theatre of Midwifery, 68, Berners Street, Oxford Street, on Monday morning the 7th of October, at half past ten; and his Evening Course at seven.

Mr. GUTHRIE, Deputy-Inspector of Hospitals, will commence his Course of Lectures on the Principles and Practice of Medical and Operative Surgery on the first Wednesday in October, at Nine o'clock in the Morning, at his house, No. 2, Berkeley Street, Berkeley Square. To be continued Mondays, Wednesdays, and Fridays.—The Operations in Surgery referred to in the Lectures, will be shewn at the York Hospital, Chelsea, on the Friday Mornings, and the Treatment illustrated by such Cases as may be in the Hospital.—Medical Officers of the Navy and Army will be admitted to attend these Lectures gratis, on obtaining a recommendation to that effect from the Heads of their respective Departments.

Dr. Merriman and Dr. Ley will deliver a Course of Lectures on Midwifery and the Diseases of Women and Children, at the Middlesex Hospital, during the Autumn.—The Introductory Lecture will be read on Monday, October 14, at half-past Ten o'clock.

---

### Meteorological Register for August, 1816.

*Kept at Harwich, in Essex.*

By JOHN BAILEY, Surgeon, &c.

| Moon's Age. | | Atmospherical Pressure. Morn. | Ev. | Temp. | Wind. | Remarks. | General Statement of Diseases. |
|---|---|---|---|---|---|---|---|
| | 1 | 29 5 | 29 6 | 65 | NW | Rain, dull | |
| | 2 | 6½ | 7 | 66 | W | Rain | Intermittentes. |
| | 3 | 7 | 7½ | 67 | NW | | |
| | 4 | 7½ | 7 | 71 | NW | | Ophthalmia |
| | 5 | 7 | 7 | 68 | SE | Rain | |
| | 6 | 8 | 7½ | 68 | W | ditto | Scarlatina |
| | 7 | 7½ | 7½ | 69 | SW | ditto | |
| ○ | 8 | 6 | 6 | 73 | W | ditto | |
| | 9 | 6 | 7 | 70 | W | ditto | |
| | 10 | 8 | 9 | 65 | NW | Dull | |
| | 11 | 9½ | 9½ | 68 | W | Rain and dull | |
| | 12 | 9½ | 9½ | 61 | W | Rain and misty | |
| | 13 | 9 | 8 | 71 | W | Very fine | |
| | 14 | 7 | 5 | 65 | W | Rain, viol. shower | |
| | 15 | 4 | 4 | 69 | SW | ditto | |
| ☾ | 16 | 4½ | 4½ | 68 | W | Rain | |
| | 17 | 5 | 7½ | 64 | W | ditto | |
| | 18 | 8 | | 64 | N | ditto | |
| | 19 | 30 | | 65 | N | Dull | |
| | 20 | | | 62 | N | Thick fog | |
| | 21 | ½ | ½ | 64 | SE | Fine and clear | |
| | 22 | | | 67 | N | Fog | |
| ● | 23 | | | 69 | NE | Dull | |
| | 24 | | | 64 | NE | ditto | |
| | 25 | | | 68 | N | ditto | |
| | 26 | | | 63 | N | ditto | |
| | 27 | | | 57 | N | ditto | |
| | 28 | | | 64 | S | Fine and clear | |
| ☽ | 29 | | 29 8½ | 62 | W | Fog | |
| | 30 | 29 7½ | 5½ | 42 | N | Cloudy | |
| | 31 | 2 | 2 | 54 | E | Stormy | |

Notwithstanding the extraordinary wetness of the present month, the list of diseases has not increased. Intermittents of the tertian kind prevail amongst the labouring class of the community, and pulmonary attacks, combined with typhoid symptoms, have, in many instances, assumed a formidable character.—An elderly woman, and a delicate and weakly man, fell victims to the disease. Pneumonia, in its genuine form and character, is a most severe malady, and shakes, to its very foundation, the soundest constitution; but, compounded with symptoms which are the offspring of a debile habit, it becomes as serious a calamity as any that visits the frail frame of human nature, and puts to the test, the best energies of him whose lot it may be to combat its baneful influence. In the hyemal quarter of 1811, your Reporter was a spectator of the mortality which this form of Pneumonia occasioned in this neighbourhood; he recollects, with painful sensations, the frequency of its occurrence among his best friends, its insidious attack, its painless progress, and resistless termination. He remembers likewise, with equal regret, the dark shadows of doubt and suspicion that hovered over the minds of surviving relatives, when death broke down the hopes and assurances of recovery, and set at nought all that the best directed measures could accomplish.—*Vide* New Medical and Physical Journal, Vol. I. page 476.

---

DEATHS.—William Groote, M. D. Apothecary to the Duchess of York.—Dr. Harwood, Physician to the Salop Infirmary.—Richard Atkinson, M. D. of Jermyn Street, St. James's.

